# Downregulation of Bcl-2 Expression by miR-34a Mediates Palmitate-Induced Min6 Cells Apoptosis

**DOI:** 10.1155/2014/258695

**Published:** 2014-04-14

**Authors:** Xiaojie Lin, Hongyu Guan, Zhimin Huang, Juan Liu, Hai Li, Guohong Wei, Xiaopei Cao, Yanbing Li

**Affiliations:** Department of Endocrinology and Diabetes Center, The First Affiliated Hospital of Sun Yat-sen University, 58 Zhongshan Road II, Guangzhou, Guangdong 510080, China

## Abstract

Recent studies have demonstrated that the expression of miR-34a is significantly upregulated and associated with cell apoptosis in pancreatic **β**-cell treated with palmitate. Nevertheless, the underlying detailed mechanism is largely unknown. Here, we showed that miR-34a was significantly induced in Min6 pancreatic **β**-cell upon palmitate treatment. Elevated miR-34a promoted Min6 cell apoptosis. Intriguingly, ectopic expression of miR-34a lowered the expression of Bcl-2, an antiapoptotic protein. Luciferase reporter assay indicated the direct interaction of miR-34a with the Bcl-2 3′-UTR. Moreover, downregulated expression of Bcl-2 induced by palmitate could be restored by inhibition of miR-34a. We conclude that direct suppression of Bcl-2 by miR-34a accounts for palmitate-induced increased apoptosis rate in pancreatic **β**-cell.

## 1. Introduction

Type 2 diabetes (T2DM) is a global public health problem and the number of affected individuals is increasing at an alarming rate [1, 2]. The pathogenesis of T2DM is characterized by a progressive insulin resistance with eventual insulin deficiency [3]. Both environmental and genetic factors are known to contribute to the development of the disease. A feedback loop exists between insulin sensitivity and insulin secretion under normal conditions. Thus, insulin secretion increases to maintain normal glucose concentration in the face of insulin resistance and/or decreasing *β*-cell mass [4, 5]. T2DM is caused when the *β*-cell compensatory response cannot be sustained [6]. Although exact prodiabetic mechanisms are still largely unknown, it has been implicated that increased serum level of free fatty acids (FFAs), alone or combined with hyperglycemia, may contribute to the pathophysiology of the disease [7]. Accumulating evidences have demonstrated that chronic high levels of circulating FFAs play a pivotal role in *β*-cell failure characterized with impaired glucose-stimulated insulin secretion (GSIS) and enhanced apoptotic level [8]. Many signaling factors, such as sterol regulatory element-binding protein (SREBP)-1c, farnesoid X receptor (FXR), and forkhead box-containing protein O (FoxO) 1, have been identified to be associated with FFAs-induced *β* cell dysfunction [9, 10]. Thus, delineating the molecular mechanisms underlying FFA-triggered pancreatic *β*-cell apoptosis would put new insights into our understanding of T2DM and open new avenues for therapy.

Two major pathways, the “extrinsic” (death receptor-induced) and “intrinsic” (Bcl-2-regulated or mitochondrial) pathways, have been demonstrated to be contributing significantly to apoptosis. The B-cell lymphoma-2 (Bcl-2) family is closely related to the intrinsic pathway of apoptosis [11]. Bcl-2 family proteins are a large family, which may either facilitate cell survival (Bcl-2, Bcl-XL, Bcl-w, and others) or promote cell death (Bax, Bak, Bad, and others) [12]. Bcl-2, one of antiapoptosis genes, was reduced significantly when treated with fatty acid [13, 14]. Accumulated evidence showed that FFA-induced pancreatic *β*-cell apoptosis was accompanied by downregulating the apoptosis inhibitor Bcl-2 expression [15, 16]. Moreover, Bcl-2 overexpression does prevent *β* cell death induced by staurosporine, FasL, and some intrinsic signals [11].

Many endeavors have been made to elucidate the mechanisms that participate in dysregulation of genes in response to palmitate in *β*-cells. Besides transcription factors, such as the forkhead box O (FOXO) transcription factor FOXO1, microRNAs (miRNAs) have been shown to represent another layer of gene regulation in palmitate-induced alteration of the gene expression [4, 17]. MiRNAs, endogenous noncoding small RNAs, bind to specific sites at the 3′-untranslated region (3′-UTR) of target genes, thereby suppressing translation and/or inducing degradation of target mRNAs [18, 19]. Deregulated expression of miRNAs is correlated with various pathological processes including cancer and diabetes [18, 20]. Recent studies by Lovis et al. have showed that miR-34a is one of the significantly upregulated miRNAs in the *β*-cell treated with palmitate [4]. Moreover, elevated expression of miR-34a enhanced the *β*-cell apoptosis [4, 21, 22]. However, the possible role of miR-34a in palmitate-induced *β*-cell apoptosis is largely unknown. Here, we found that downregulation of Bcl-2 expression by miR-34a accounts for palmitate-induced increased apoptosis of pancreatic *β*-cell.

## 2. Material and Methods

### 2.1. Cell Culture and Palmitate Treatment

Min6 pancreatic *β*-cells were maintained in DMEM medium (Gibco, Carlsbad, CA) containing 25 mM glucose supplemented with 10% fetal bovine serum (FBS), 10 nM 4-(2-hydroxyethyl)-1-piperazineethanesulfonic acid (HEPES), 2 mM L-glutamine, 100 Unit/mL penicillin, 100 *μ*g/mL streptomycin, and 50 *μ*M *β*-mercaptoethanol at 37°C in 5% CO_2_. A stock solution (100 nM) of palmitate (Sigma, St. Louis, MO) was prepared by dissolving the fatty acid in 50% ethanol and then diluted in culture medium with 0.5% fatty acid-free BSA (Sigma, St. Louis, MO) to a final concentration of 1.0 mM. The palmitate/BSA conjugates were administered on culture cells.

### 2.2. Plasmids and Constructs

Cloning of the mouse Bcl-2 3′-UTR containing miR-34a binding site was performed by PCR using the following primers: forward: 5′-gcgccgcggtcgacaaacctgccccaaac-3′; reverse: 5′-gcgctgcagacgtttcttggcaattcctg-3′. The PCR product was then cloned into a modified pGL3 control vector where SacII and PstI sites were introduced into the original Xba I site downstream of the luciferase gene. To mutate the binding sites within the 3′-UTR, QuikChange site-directed mutagenesis kit from Stratagene (La Jolla, CA) was used according to the instruction manual. Cloning of the mouse Bcl-2 ORF was performed by PCR using following primers: forward: 5′-ataggtaccatggcgcaagccgggagaac-3′; reverse: 5′-cggtctagatcacttgtggcccaggtatg-3′. The PCR product and the expression vector pcDNA3 (Invitrogen, Carlsbad, CA) were cut with KpnI and XbaI and ligated to generate the construct pcDNA3-M-Bcl-2.

### 2.3. SYBR Green Quantitative PCR Analysis

Real-time PCR was performed using SYBR Premix Ex Taq (Takara, Dalian, China) in an ABI7500 system (Applied Biosystems, Carlsbad, CA, USA). Bulge-loop miRNA qPCR primers for miR-34a were synthesized by RiboBio (Guangzhou, China). U6 was used as the internal control.

### 2.4. Western Blotting (WB)

Western blotting was performed according to a standard method as described previously [23]. The following primary antibodies were used: anti-Bcl-2 and anti-cleaved-caspase-3 (Cell Signaling, Beverly, MA), and anti-*α*-tubulin (Sigma-Aldrich, St Louis, MO).

### 2.5. Transient Transfection

MiRNA mimic, miRNA inhibitor, and their corresponding control oligonucleotides (purchased from RiboBio, Guangzhou, China) were transfected using Lipofectamine 2000 following the manufacturer's instruction (Invitrogen, Carlsbad, CA). The Bcl-2 siRNA transfection was performed by following the same procedure. Sense-strand sequences of Bcl-2 siRNAs are 5′-AAGUACAUACAUUAUAAGCUG-3′ and 5′-GUACAUCCAUUAUAAGCUG-3′.

### 2.6. Apoptosis Assay

The number of apoptotic cells was evaluated by scoring cells displaying pycnotic nucleus and/or fragmented nucleus visualized with Hoechst 33342 as previously described [24].

### 2.7. Caspase 3 Activity Assay

A commercial caspase 3 activity assay kit (Roche, Mannheim, Germany) was used to measure the intracellular caspase 3 activity according to manufacturer's instruction.

### 2.8. Reporter Assay

For the reporter assay, cells were seeded in triplicate in 24-well plates and allowed to settle for 24 h. Indicated plasmids and oligonucleotides were transfected into the cells using the Lipofectamine 2000 reagent (Invitrogen, Carlsbad, CA). Forty-eight hours after transfection, Dual-Luciferase reporter assays were performed according to the manufacturer's instructions (Promega, Madison, WI). Results are presented as means ± standard deviation (SD) of three independent experiments performed in triplicate.

### 2.9. Statistical Analysis

All statistical analyses were carried out using the SPSS 11.0 statistical software package. All values represent at least three independent experiments and are expressed as the means ± SD. The differences between two experimental conditions were compared on a one-to-one basis using Student's* t-*tests. Statistical significance within groups was analyzed using 1-way ANOVA, followed by a Dunnett's post hoc test. *P* < 0.05 was considered statistically significant.

## 3. Results

### 3.1. MiR-34a Is Upregulated and Is Associated with Cell Apoptosis in *β* Cell in Response to Palmitate Treatment

**Figure 1:**
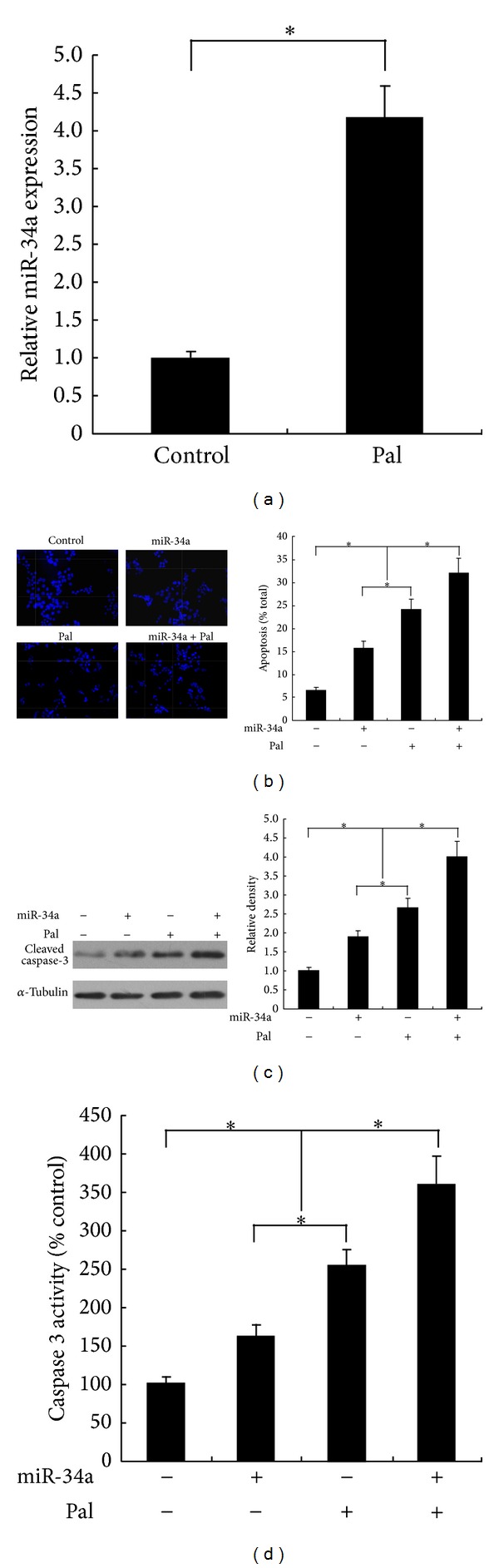
MiR-34a involves in palmitate-induced Min6 cells apoptosis. (a) miR-34a expression level was elevated in response to palmitate treatment. (b) ectopic expression of miR-34a enhanced Min6 cell apoptosis. Cells transduced or not with miR-34a were treated with palmitate (500 *μ*M) or not for 2 days. The apoptotic rate of cells was determined by scoring the cells displaying pycnotic nucleus and/or fragmented nucleus. Cleaved caspase-3 expression (c) and caspase-3 activity (d) were carried out to confirm the proapoptotic role of miR-34a in Min6 cells. For (a), (b), (c) and (d), data are shown as means ± SD of three independent experiments. *P < 0.05.

We first assessed whether palmitate can induce miR-34a expression in Min6 pancreatic *β* cell. As shown in Figure 1(a), the expression level of miR-34a was significantly elevated by palmitate treatment in Min6 cells. Next, we investigated whether change in the level of miR-34a induced by palmitate was able to influence apoptotic rate of the cells. As shown in Figure 1(b), ectopic expression of miR-34a increased the number of dying cells. As the caspase-3 activity is a key marker of apoptosis, the expressions of cleaved caspase-3 and caspase-3 activity were investigated to confirm the proapoptotic role of miR-34a. As shown in Figures 1(c) and 1(d), overexpression of miR-34a increased the expression of cleaved caspase-3 and caspase-3 activity. Together, these data suggested that upregulated miR-34a contributes to proapoptotic effects of palmitate on pancreatic *β* cell.

### 3.2. Bcl-2 Is Directly Targeted by miR-34a

**Figure 2:**
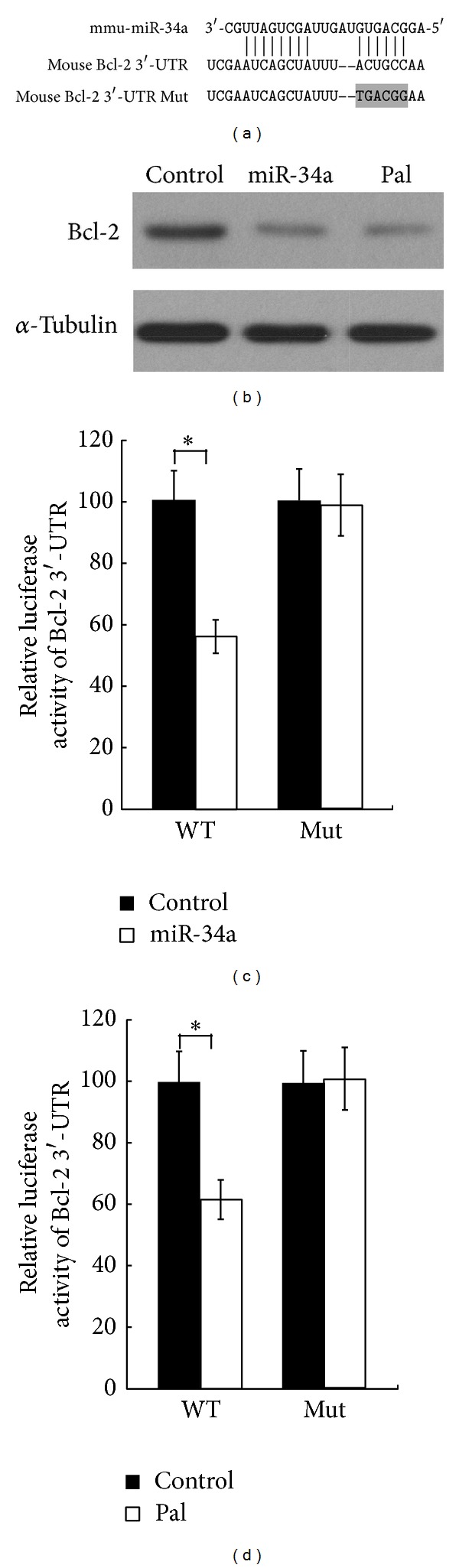
MiR-34a directly targets Bcl-2. (a) miR-34a binding site in the 3′-UTR and mutated site in 3′-UTR of Bcl-2 in the luciferase reporter. (b) Ectopic expression of miR-34a or palmitate treatment significantly suppressed the protein level of Bcl-2 in Min6 cells. (c) miR-34a inhibited the Bcl-2 expression by interacting with the 3′-UTR of Bcl-2. Min6 cells were cotransfected with the luciferase reporter vector containing wild-type or mutated 3′-UTR and miR-34a mimic or control oligonucleotides. (d) The effects of palmitate treatment on the luciferase activity of reporter vector containing wild-type or mutated 3′-UTR. For (c) and (d), data are shown as means ± SD of three independent experiments *P < 0.05.

We next determined how miR-34a influences the *β* cell apoptosis. Bcl-2, an antiapoptotic protein involved in *β* cell apoptosis, is of specific interest as the computational tool (TargetScan [25] http://www.targetscan.org/) predicted a miR-34a-binding site within the Bcl-2 3′-UTR (Figure 2(a)). To verify the potential targeting of Bcl-2 by miR-34a, we measured the protein level of Bcl-2 in miR-34a transfected Min6 cells. As shown in Figure 2(b), the expression of Bcl-2 was significantly suppressed by miR-34a transfection. To further confirm that the suppression of Bcl-2 expression is directly miR-34a-driven, we generated a luciferase construct containing the Bcl-2 3′-UTR. Meanwhile, a construction with the mutation at the putative binding site (Figure 2(a)) was also generated as control. Cotransfection of the wild-type Bcl-2 3′-UTR and miR-34a mimic led to a significant inhibition of luciferase activity (Figure 2(c)). In contrast, no significant alteration of luciferase activity was detected in cells transfected with mutated construct. Consistently, palmitate treatment also suppressed the luciferase activity of the wild-type Bcl-2 3′-UTR and had no effect on mutated construct (Figure 2(d)). Taken together, these data suggest that miR-34a suppresses Bcl-2 expression via directly binding to its 3′-UTR.

### 3.3. Pretreatment of miR-34a Inhibitor Antagonizes the Effects of Palmitate on Pancreatic *β* Cell

**Figure 3:**
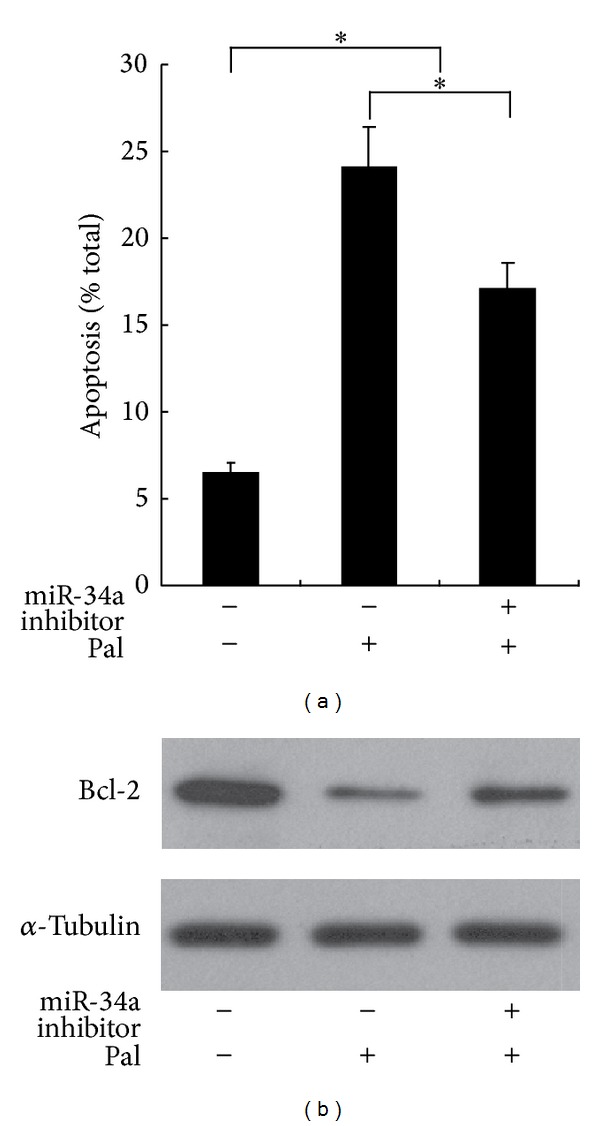
Pretreatment of miR-34a inhibitor antagonizes the effects of palmitate on pancreatic *β* cell. Min6 cells were transduced with either miR-34a inhibitor or control oligonucleotides. Twenty-four hours later, indicated cells were treated with palmitate for 48 h. The apoptotic rate of cells was determined by scoring the cells displaying pycnotic nucleus and/or fragmented nucleus (a) *P < 0.05. The expression levels of Bcl-2 were assessed (b).

Considering the apparent increase in the expression of miR-34a in response to palmitate, it is expected that pretreatment of miR-34a inhibitor may prevent the palmitate imposed effects. Indeed, we showed that the increased apoptotic rate and decreased Bcl-2 expression induced by palmitate in pancreatic *β* cell could be counteracted by pretreatment with miR-34a inhibitor (Figures 3(a) and 3(b)). Therefore, upregulated miR-34a expression participates in the palmitate-induced effects on pancreatic *β* cell.

### 3.4. Bcl-2 Is Functionally Related to the Effect of miR-34a in Response to Palmitate

**Figure 4:**
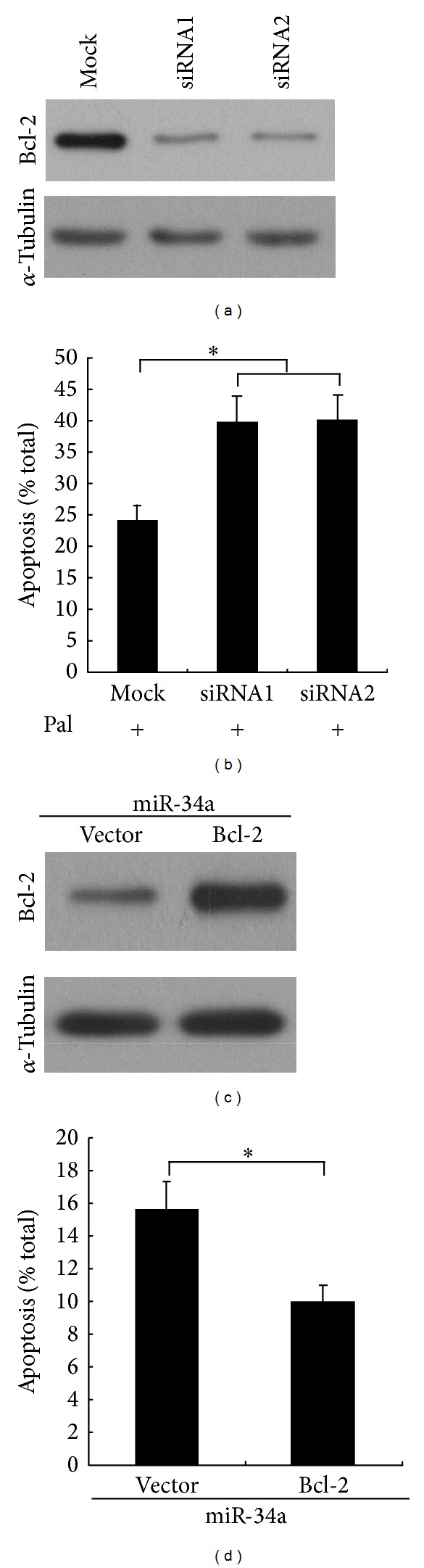
Bcl-2 is functionally related to the effect of miR-34a in response to palmitate. (a) Min6 cells transfected with specific siRNAs against mouse Bcl-2. WB assay were performed to confirm the effect of siRNAs. *α*-Tubulin was used as loading control. (b) Min6 cells were transfected with specific siRNAs against mouse Bcl-2. The cells were cultured for 48 h in DMEM containing 1 mM palmitate. The number of apoptotic cells was evaluated by scoring cells displaying pycnotic nucleus and/or fragmented nucleus visualized with Hoechst 33342. (c) Reexpression of Bcl-2 with the expression plasmid pcDNA3-M-Bcl-2 in the miR-34a-overexpressing Min6 cells. WB assay was performed to confirm the effect of Bcl-2 overexpression. *α*-Tubulin was used as loading control. (d) Reexpression of Bcl-2 partially antagonized the miR-34a-induced cell apoptosis. For (b) and (c), data are means ± SD of triplicate assays in three independent experiments *P < 0.05.

To confirm the functional role of Bcl-2 in pancreatic *β* cell, inhibition of Bcl-2 expression by siRNAs was conducted. WB assays were performed and confirmed that Bcl-2 expression was significantly silenced (Figure 4(a)). Then we investigated the effect of knockdown of Bcl-2 on *β* cell apoptosis. As expected, data presented in Figure 4(b) showed that inhibition of Bcl-2 caused increased Min6 cell apoptosis. Moreover, reexpression of Bcl-2 in miR-34a-expressing Min6 cells (Figure 4(c)) partially antagonized the miR-34a-induced apoptosis. Taken as a whole, these data indicate that Bcl-2 is a functionally relevant target of miR-34a's effect in response to palmitate treatment.

## 4. Discussion

It has been previously demonstrated that Bcl-2 expression is suppressed during palmitate-induced apoptosis [26] and miR-34a expression was upregulated in response to palmitate treatment [4]. Furthermore, analysis of the 3′-UTR of Bcl-2 gene shows potential miR-34a binding site. This raised the possibility that suppression of Bcl-2 in apoptotic cells may be regulated by miR-34a. We evaluated this hypothesis in Min6 pancreatic *β* cell, where we showed that Bcl-2 is directly targeted by miR-34a. MiR-34a-induced suppression of Bcl-2 is associated with palmitate-associated pancreatic *β* cell apoptosis.

T2DM is a metabolic disorder that is driven by the interaction between genetic and acquired factors. Overweight and lack of exercise are lifestyle factors associated with T2DM [27]. The impact of ectopic fat and lipotoxicity on pancreatic *β*-cell dysfunction and insulin resistance in humans has been paid great attention. Accumulated evidence shows that lipotoxic stress-induced *β*-cell death (lipotoxicity) is a key contributor to the development of T2DM by inducing insulin resistance [28]. Lipotoxicity is caused by circulating free fatty acids increased, changes in lipoprotein profiles and body fat distribution. It is widely accepted that a sustained exposure to fatty acids impairs glucose-stimulated insulin secretion (GSIS), promotes apoptosis, and alters insulin gene expression in vitro and in vivo [29]. Bcl-2, an antiapoptotic Bcl-2 family member, is a crucial controller of the mitochondrial pathway of pancreatic *β*-cell apoptosis induced by lipotoxicity [30]. Although previous studies suggest that the suppression of Bcl-2 is involved in lipotoxicity induced pancreatic *β* cell apoptosis, the mechanisms involved in the decreased expression of Bcl-2 in response to palmitate remains to be clarified.

In researching the mechanism of palmitate-induced downregulated expression of Bcl-2, we noted that miR-34a, as palmitate, can dramatically induce its expression. It has been demonstrated that miR-34a was involved in various cell apoptosis conditions, including pancreatic beta cells. Roggli et al. have demonstrated that anti-miR-34a can protect pancreatic *β* cell from proinflammatory cytokines-induced cell death and miR-34a-triggered Bcl-2 downregulation might be associated with cytokine-mediated *β* cell apoptosis [20]. Moreover, miR-34a can lead to apoptosis via suppression of SIRT1 in cells [31]. If Bcl-2 was inhibited by miR-34a overexpression, it is likely that the effect of palmitate on *β* cell apoptosis is mediated through suppression of Bcl-2 by miR-34a. Here, we clearly verified that Bcl-2 is a target of miR-34a. An in silico approach identified a potential miR-34a binding site within the 3′-UTR of Bcl-2 gene. Reporter gene analysis confirmed the prediction that miR-34a directly repress Bcl-2. Furthermore, the protein level of Bcl-2 is significantly suppressed by ectopic expression of miR-34a. Intriguingly, anti-miR-34a pretreatment was able to attenuate palmitate-induced Bcl-2 downregulation, as well as *β* cell apoptosis, strongly indicating the functional role of miR-34a in the *β* cell in response to lipotoxicity. We also investigated the importance of Bcl-2 for palmitate-induced cell apoptosis in Min6 pancreatic *β* cell lines by loss- and gain-of-function experiments.

Given that one specific miRNA can regulate a group of target genes, together with the fact that repressing Bcl-2 could not fully recapitulate miR-34a phenotype in pancreatic *β* cell, it is expected that other target genes regulated by miR-34a are involved in palmitate-induced *β* cell dysfunction. Future work is still needed in the area of defining possible target genes of miR-34a that contribute to apoptosis of *β* cell exposed to palmitate.

In conclusion, suppression of Bcl-2 by miR-34a accounts for palmitate-induced increased apoptotic rate in pancreatic *β* cell. Our findings reveal a new mechanistic aspect of the proapoptotic effect of palmitate on pancreatic *β* cell and raise the possibility of influencing miR-34a as a therapeutic strategy for diabetes.

## References

[1] Wild S, Roglic G, Green A, Sicree R, King H (2004). Global prevalence of diabetes: estimates for the year 2000 and projections for 2030. *Diabetes Care*.

[2] Ballav C, Gough SC (2013). Safety and efficacy of sitagliptin-metformin in fixed combination for the treatment of type 2 diabetes mellitus. *Clinical Medicine Insights: Endocrinology and Diabetes*.

[3] Kim YN, Kim S, Kim IY (2013). Transcriptomic analysis of insulin-sensitive tissues from anti-diabetic drug treated Zdf rats, a T2Dm animal model. *PLoS ONE*.

[4] Lovis P, Roggli E, Laybutt DR (2008). Alterations in microRNA expression contribute to fatty acid-induced pancreatic *β*-cell dysfunction. *Diabetes*.

[5] Weir GC, Bonner-Weir S (2004). Five of stages of evolving *β*-cell dysfunction during progression to diabetes. *Diabetes*.

[6] Prentki M, Nolan CJ (2006). Islet *β* cell failure in type 2 diabetes. *Journal of Clinical Investigation*.

[7] Wilding JPH (2007). The importance of free fatty acids in the development of type 2 diabetes. *Diabetic Medicine*.

[8] Yaney GC, Corkey BE (2003). Fatty acid metabolism and insulin secretion in pancreatic beta cells. *Diabetologia*.

[9] Shao S, Yang Y, Yuan G, Zhang M, Yu X (2013). Signaling molecules involved in lipid-induced pancreatic beta-cell dysfunction. *DNA and Cell Biology*.

[10] Cusi K (2010). The role of adipose tissue and lipotoxicity in the pathogenesis of type 2 diabetes. *Current Diabetes Reports*.

[11] Thomas HE, McKenzie MD, Angstetra E, Campbell PD, Kay TW (2009). Beta cell apoptosis in diabetes. *Apoptosis*.

[12] Lupi R, Dotta F, Marselli L (2002). Prolonged exposure to free fatty acids has cytostatic and pro-apoptotic effects on human pancreatic islets: evidence that *β*-cell death is caspase mediated, partially dependent on ceramide pathway, and Bcl-2 regulated. *Diabetes*.

[13] Roset R, Ortet L, Gil-Gomez G (2007). Role of Bcl-2 family members on apoptosis: what we have learned from knock-out mice. *Frontiers in Bioscience*.

[14] Shimabukuro M, Wang M-Y, Zhou Y-T, Newgard CB, Unger RH (1998). Protection against lipoapoptosis of *β* cells through leptin-dependent maintenance of Bcl-2 expression. *Proceedings of the National Academy of Sciences of the United States of America*.

[15] Liang H, Zhong Y, Zhou S, Li QQ (2011). Palmitic acid-induced apoptosis in pancreatic *β*-cells is increased by liver X receptor agonist and attenuated by eicosapentaenoate. *In Vivo*.

[16] Liu L, Wang Y, Wang L (2013). Exendin-4 protects murine pancreatic beta-cells from free fatty acid-induced apoptosis through PI-3K signaling. *Endocrine Research*.

[17] Li Y, Xu X, Liang Y (2010). miR-375 enhances palmitate-induced lipoapoptosis in insulin-secreting NIT-1 cells by repressing myotrophin (V1) protein expression. *International Journal of Clinical and Experimental Pathology*.

[18] Huang B, Qin W, Zhao B (2009). Microrna expression profiling in diabetic Gk rat model. *Acta Biochimica et Biophysica Sinica*.

[19] Klein D, Misawa R, Bravo-Egana V (2013). Microrna expression in alpha and beta cells of human pancreatic islets. *PLoS ONE*.

[20] Roggli E, Britan A, Gattesco S (2010). Involvement of microRNAs in the cytotoxic effects exerted by proinflammatory cytokines on pancreatic *β*-cells. *Diabetes*.

[21] Locke JM, Allen Lango H, Harries LW (2013). A rare SNP in pre-mir-34a is associated with increased levels of mir-34a in pancreatic beta cells. *Acta Diabetologica*.

[22] Chen F, Hu S-J (2012). Effect of microRNA-34a in cell cycle, differentiation, and apoptosis: a review. *Journal of Biochemical and Molecular Toxicology*.

[23] Guan H, Liu L, Cai J (2011). Sphingosine kinase 1 is overexpressed and promotes proliferation in human thyroid cancer. *Molecular Endocrinology*.

[24] Abderrahmani A, Niederhauser G, Favre D (2007). Human high-density lipoprotein particles prevent activation of the JNK pathway induced by human oxidised low-density lipoprotein particles in pancreatic beta cells. *Diabetologia*.

[25] Lewis BP, Burge CB, Bartel DP (2005). Conserved seed pairing, often flanked by adenosines, indicates that thousands of human genes are microRNA targets. *Cell*.

[26] Choi S-E, Kim H-E, Shin H-C (2007). Involvement of Ca^2+^-mediated apoptotic signals in palmitate-induced MIN6N8a beta cell death. *Molecular and Cellular Endocrinology*.

[27] Lencioni C, Lupi R, Del Prato S (2008). *β*-cell failure in type 2 diabetes mellitus. *Current Diabetes Reports*.

[28] Qi Y, Chen J, Lay A, Don A, Vadas M, Xia P (2013). Loss of sphingosine kinase 1 predisposes to the onset of diabetes via promoting pancreatic beta-cell death in diet-induced obese mice. *FASEB Journal*.

[29] Cusi K (2010). The role of adipose tissue and lipotoxicity in the pathogenesis of type 2 diabetes. *Current Diabetes Reports*.

[30] Gurzov EN, Eizirik DL (2011). Bcl-2 proteins in diabetes: mitochondrial pathways of *β*-cell death and dysfunction. *Trends in Cell Biology*.

[31] Yamakuchi M, Ferlito M, Lowenstein CJ (2008). miR-34a repression of SIRT1 regulates apoptosis. *Proceedings of the National Academy of Sciences of the United States of America*.

